# Effects of socio-economic factors on research over systemic sclerosis: an analysis based on long time series of bibliometric data

**DOI:** 10.1186/s13023-021-02149-w

**Published:** 2021-12-20

**Authors:** Wei Guo, Zeyu Zhou, Yinhe Liang, Chuanhui Xu, Lin Zeng, Zhiyong Dong, Rong Mu

**Affiliations:** 1 Department of Rheumatology and Immunology, Peking University Third Hospital, 49 North Hua Yuan Road, Hai Dian District, Beijing, 100191 China; 2 School of Economics, Peking University, 5 Yi He Yuan Road, Hai Dian District, Beijing, 100871 China; 3 School of Economics, Central University of Finance and Economics, Beijing, China; 4 Department of Rheumatology, Allergy and Immunology, Tan Tock Seng Hospital, Singapore, Singapore; 5 Research Center of Clinical Epidemiology, Peking University Third Hospital, Beijing, China

**Keywords:** Systemic sclerosis, Research output, Bibliometrics

## Abstract

**Background:**

Systemic sclerosis (SSc) is a rare detrimental disease warranting global research efforts. Evaluating how socio-economic factors impact country research output on SSc could help to identify solutions advancing research.

**Methods:**

Publication production on SSc during 1969–2018 and data for structural and policy factors for WHO member countries were collected from public sources. Associations between SSc research output and country-level factors were investigated through panel regression. Difference-in-differences analysis further assessed the causal effects of rare disease legislation.

**Results:**

SSc publications demonstrated exponential growth (*r* = 0.9410, as against an *r* = 0.8845 after linear adjustment), but were concentrated in high-income countries (HICs). Ten countries, nine of which were HICs, published 12,261 (77.5%) SSc publications but another 87 countries produced none. Gross domestic products (GDP), population and expenditure on research and development were positively associated with SSc publications (*p* < 0.001). Higher health expenditure was only found to be associated with increased SSc publications in HICs (*p* < 0.001). Rare disease legislation increased annual publication production by 62.8% (95% CI 0.390–0.867; *p* < 0.001) averagely. In middle-income countries (MICs), the effect was especially swift and lasting. No significant impact was found with GDP per capita, female percentage, and political indicators.

**Conclusions:**

SSc research output increased over time with substantial country disparities. Effective health policies facilitating research should be expanded especially among MICs to accelerate research advancement.

**Supplementary Information:**

The online version contains supplementary material available at 10.1186/s13023-021-02149-w.

## Introduction

Systemic sclerosis (SSc) is a rare autoimmune disease affecting 9.3–341 cases per million people [1, 2]. The disease is characterized with systemic vasculopathy, immune dysfunction and organ fibrosis causing multiple devastating complications. Current treatment of SSc are mainly directed at symptom control and no curing therapies are available for universal problems in SSc patients including skin fibrosis and interstitial lung disease. As a result, SSc has the highest mortality among rheumatic diseases, more than half of patients ultimately dying directly of the illness or of related complications [3, 4].

Research on SSc has been challenging because of the multifaceted and rare nature of the disease. With SSc patients populated sparsely without specific geographical or racial patterns [5], it is important to promote SSc research worldwide, which could not only translate to better diagnosis and interventions for all SSc patients, but also promoted domestic evidence-based management for patients in a certain country [6]. Assessing how socio-economic factors affect research output might help to identify measures advancing SSc research,

Previous studies have reported research insufficiency in developing countries on other medical issues, including obesity [7], biliary atresia [8] and neurosurgery [9]. A recent study analyzing clinical studies on SSc also identified research concentration in a few developed countries [10]. Contrastingly, several studies revealed that gross domestic production (GDP), GDP per capita, and population were not necessarily associated with research output [11–13]. Stringent analysis assessing associations between socio-economic indicator and SSc research output has not been conducted, which is critical for identifying promoting or handicapping factors.

In addition to find out structural factors conferring research advantages/disadvantages, it is even more important to identify effective national policies to address research inequality. Rodriguez-Granillo et al. [14] found that % GDP expenditure in research and development or health were associated with research output on cardiovascular medicine. On childhood immunization, Wiysonge et al. [11] revealed that only private health expenditure was associated with research output using multivariable regression. Nonetheless, effects of government expenditure might be different concerning SSc, considering that rare disease is usually marginalized in healthcare and research framework. Another policy factor of interest is legislation aimed for welfare of patients with rare diseases. Rare diseases are diseases with prevalence under certain threshold, which varies across countries with an average between 400 and 500 cases per million people [15]. Thus SSc is a rare disease by definition and research over it could be influenced by rare disease legislation, yet no assessment has been conducted. Though has an increase of overall orphan drug approval been described since the hallmarking 1983 Orphan Drug Act [16], such increase might be merely reflection of general scientific development instead of resulting from legislation progress. Validating the concept that rare disease legislation might affect scientific output is of critical importance, especially nowadays when countries including China are still lagging in rare disease legislation.

In this study, we aimed to quantitatively describe the global SSc academic output and evaluate the impact of country-level factors to explore solutions supporting research conduction on SSc.

## Methods

### Data collection

Scopus was searched on October 15, 2019 to retrieve SSc journal publications for its wide indexing of journals [17]. Publications with “systemic sclerosis” or “scleroderma” but not “localised/localized scleroderma” in titles or keywords were identified as SSc publications. The search was limited to the period from January 1, 1969 to December 31, 2018 to learn about SSc publications produced in the last half century. We did not include publications before 1969 in consideration of quality and availability for bibliometric and socio-economic data. Publications after 2019 were also excluded to avoid data bias caused by database updating. Retracted publications, letters, editorials, and erratum were excluded.

Data on country level structural and policy factors were collected from different sources as listed in Additional file 1: Table S1 (including definitions and data coverage). Structural indicators included GDP [18], population [18], GDP per capita, female population percentage [19], and political measures including voice and accountability, government effectiveness, and political stability, and absence of violence/terrorism [20]. Country stratification assigned by World Bank based on Gross national income per capita was used as country grouping criteria but not included as a variable in the regression analysis. Briefly, countries were classified as high-income countries (HICs), middle-income countries (MICs) or low-income countries (LICs) using the gross national income per capita threshold of 12,375$ and 1026$ [19]. Country-level policy factors examined in the present study included investment into related areas, research and development and health (presented as percentage of GDP) [19, 21] as well as the status of rare disease legislation in 2017 and year of commencement from the revalidated policy review [22]. It should be noted that during 2000–2017, all indicators are with available data.

## Research output determination

Global annual production and a list of contributing countries of SSc publications were exported from Scopus. Annual numbers of publications in the area of health and life sciences were exported similarly for comparison. Bibliometric information of SSc publications including publication years, affiliations, and correspondence addresses was extracted to generate SSc publication production of given countries in the given time. The attribution of publications to countries was based on affiliations and correspondence addresses, with one authorship to each contributing country. Publications without country information were dropped in country-level analysis.

### Outcome and explanatory variables

SSc scientific output was measured by the number of SSc publications. Studied country-level factors included economic, demographic, political, and policy factors as stated above. Depending on models fitted, two different variables were used to represent rare disease legislation. The first is a time-invariant categorical variable $L_{i}$ for which one indicating with rare disease legislation and zero indicating otherwise in 2017. The second is a time-variant variable $L_{it}$ to indicate the status of rare disease legislation in a given country and given year, with value one assigned to countries after 1 year from rare disease legislation commencement, and zero to other conditions. The use of $L_{it}$ allows for more accurate estimation of legislation effect. Other explanatory variables were all time-variant and continual. To reduce skewness and stabilize the variance, SSc publications, GDP, GDP per capita, and population were transformed to ln (SSc publications + 1), ln GDP, ln GDP per capita, and ln population in regression analysis; one was added to SSc publications before the logarithmic transformation to avoid zero values. Coefficients derived from regression assessing ln of SSc publications represented the increased percentage of SSc publications with one-unit change of the explanatory variable given *ceteris paribus*.

### Sample characteristics

WHO member countries found with available data were included in corresponding analysis. Totally, 1442 observations from 132 countries were analyzed by panel regression for association analysis with all country factors on the period 2000–2017 and 7649 observations from 167 countries were included in the difference-in-differences (DID) analysis assessing legislation effect on 1969–2018 (Table 1). Countries were stratified as high, middle, and low-income countries using gross national income per capita for 2018 according to the World Bank, it should be noted that the stratification was not used as explanatory variable, thus the change of economic stratification during the studies period does not undermine our results. Half of the countries were MICs (68 [51.5%] in the 2000–2017 dataset; 89 [53.3%] in the 1969–2018 dataset). In our datasets, 59.6% (31/52) HICs have adopted rare disease legislation, mostly before 2007, while only 14 of the 89 MICs (15.7%) have rare disease legislation, mostly after 2007. None of the LICs were with rare disease legislation.

**Table 1:** Characteristics of country-year samples for regression on annual SSc publications and country-level factors

	2000–2017 dataset (No. country-years = 1442)	1969–2018 dataset (No. country-years = 7649)
Total countries, n	132	167
Annual SSc publications, mean (SD)	6.22 (18.24)	2.33 (8.06)
Population in million, mean (SD)	48.13 (158.54)	32.72 (118.49)
Female percentage of population, mean (SD)	49.96 (3.71)	50.14 (2.58)
GDP in billion 2011US$, mean (SD)	627.72 (1814.24)	314.69 (1032.69)
GDP in billion 2011US$, mean (SD)	627.72 (1814.24)	314.69 (1032.69)
Voice and accountability, mean (SD)	0.02 (0.97)	NA
Government effectiveness, mean (SD)	0.16 (0.94)	NA
Political stability and absence of violence/terrorism, mean (SD)	− 0.06 (0.90)	NA
Research and development expenditure in percentage of total GDP, mean (SD)	0.75 (0.87)	NA
Health expenditure in percentage of total GDP, mean (SD)	6.29 (2.28)	NA
Income groups		
High income, n (%)	49 (37.1%)	52 (31.1%)
Middle income, n (%)	68 (51.5%)	89 (53.3%)
Low income, n (%)	15 (11.4%)	26 (15.6%)
Rare disease legislation	45 (34.1%)	45 (26.9%)
Legislation before 1998, n (HICs/MICs/LICs)	2 (2/0/0)	2 (2/0/0)
Legislation during 1998–2007, n (HICs/MICs/LICs)	32 (28/4/0)	32 (28/4/0)
Legislation during 2008–2017, n (HICs/MICs/LICs)	11 (1/10/0)	11 (1/10/0)

Continual statistics were summarized by average values during the same period of regression analysis. Number of countries adopting rare disease legislations in given period were presented as in total and income groups. Data for world governance indicators, research and development expenditure, and health expenditure were only available over 2000–2017 and were therefore not included in the 1969–2018 regression or summarized here

*GDP* gross domestic product, *HICs* high-income countries, *LICs* low-income countries, *MICs* middle-income countries, *NA* not applicable

### Statistical analysis

*Price’s Law*, a widely used bibliometric indicator reflecting pattern of scientific production increase [23], was applied to test exponential growth in scientific output on SSc. We completed an exponential adjustment of the data, utilizing the equation $y = 8.42{\text{E}} - 34e^{0.04105*x}$, and an additional linear adjustment, using the equation $y = - 30551.40 + 15.51{*}x$.

Multivariate linear regression models were used to evaluate associations between SSc scientific output and all country-level indicators with data on the period 2000–2017 considering data availability mentioned beforehand. Impacts of GDP per capita, population, female population percentage, voice and accountability, government effectiveness, political stability and absence of violence/terrorism, research and development expenditure, health expenditure, and rare disease legislation of country $i$ with the time-invariant $L_{i}$, were assessed over countries with available data. GDP per capita, population, and GDP cannot be present in the same model due to collinearity. In an additional model, we replaced GDP per capita and population with GDP to evaluate the effect of total economy size. We included year fixed effects affecting countries equally and changing over time but not country fixed effects controlling for country inherent factors to avoid omission of $L_{i}$ coefficient due to data collinearity. Observations with missing data were dropped in both models. We used standard errors clustered to countries in all regression analyses.

DID models (M1–M3) were used to assess the effects of rare disease legislation on SSc scientific output more accurately using the DID variable $L_{it}$ with panel data spanning 1969–2018. Different from $L_{i}$ used in former models generally assessing effects of having or not having rare disease legislation until 2017, $L_{it}$ allows for more accurate evaluation. $L_{it}$ indicates the status of rare disease legislation determined on both of a given country $i$ and a given year $t$. Coefficients of $L_{it}$ represented the average effect of rare disease legislation on country SSc scientific output. GDP per capita, population, and female population, of which data were accessible over the studied period, were controlled in all three models (M1–M3). In M2 and M3, country and year fixed effects were added sequentially for control of fixed effects as stated earlier. In particular, the inclusion of country fixed effects enabled country-specific control of time-stable factors, e.g. long-term level of SSc prevalence, historical cultural background and domestic political system. Observations with other data missing were excluded from the DID analysis. Standard errors clustered to countries were used. Sensitivity analysis was done using imputed 2018 data on population and GDP per capita with data on 2017 when available.

Inference of causal effect using DID analysis is based on the assumption that without rare disease legislation, all countries included in DID analysis would have the same trends with the outcome measure [24]. This parallel trend assumption was tested by including leading dummies of the legislation variable in a supplementary model. Coefficients of the leads should not be statistically different from zero when the parallel trend assumption is satisfied. Moreover, we included lags to assess the effect of rare disease legislation over time. Leads up to 5 years before legislation and lags up to 10 years after legislation were included in the supplementary regression to testify parallel trend assumption and assess the effect dynamics of legislation.

Two-sided significance tests were used and significance was set at *p* less than 0.05. Statistical analysis was conducted using Stata 16 (Stata-Corp LP, College Station, TX). Data for temporal and geographical distribution of publications were visualized using R v4.1.

## Results

### SSc publications production increased rapidly in the new century

**Fig. 1:**
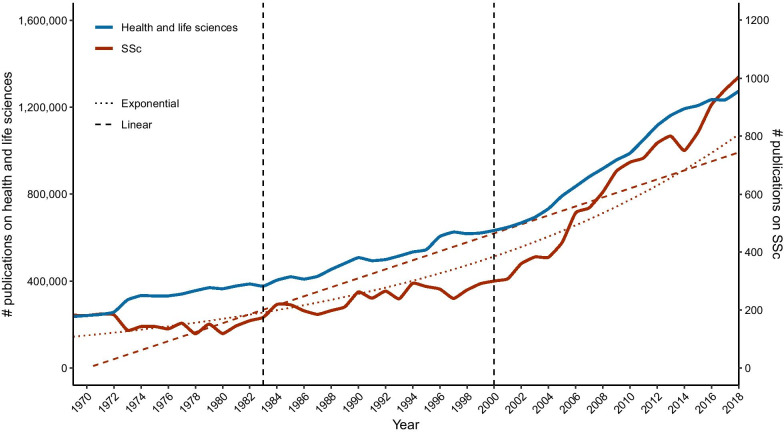
Time distribution of SSc publications. Numbers of SSc (red solid line) and health and life sciences (blue solid line) publications are shown by year during 1969–2018, which was divided into three stages according to the speed of publication accumulation. Both linear and exponential adjustment of the data was carried out to check whether production follows *Price’s Law* of exponential growth. Exponential adjustment (red dashed line): $y = 8.42E - 34e^{0.04105*x} { }\left( {r^{2} = 0.8855} \right)$. Linear adjustment (red dotted line): $y = - 30551.40 + 15.51*x \left( {r^{2} = 0.7824} \right)$. *SSc* systemic sclerosis

The literature search through Scopus retrieved 18,175 publications in the area of SSc published from January 1, 1969 to December 31, 2018. Figure 1 showed the time trend of SSc publication growth with comparison to that of total publications in the area of health and life medicines. Annual SSc publication production fluctuated under 200 (135–186, average annual growth rate: − 0.3% with SSc vs. 3.4% with whole health and life sciences) before 1983, then increased in parallel with health and life sciences, reaching an annual production of 300 (average annual growth rate: 3.2% with SSc vs. 3.1% with whole health and life sciences) until around the year of 2000. An accelerated publication of SSc literature was shown from 2000. Two-thirds of SSc publications (66.0%, 11,987/18,175) were published from 2000 to 2018. Annual production increased 3.3 folds to 1004 in 2018 compared to two folds in the whole health and life sciences area (average annual growth rate: 6.9% with SSc vs. 4.0% with whole health and life sciences). Accordingly, mathematical adjustment of SSc publications to the exponential curve creates a correlation coefficient *r* = 0.9410 compared to an *r* = 0.8845 with linear adjustment. Number of SSc publications thus better aligns with an exponential fit rather than a linear one, and therefore with the postulates of *Price’s Law*.

### SSc scientific output varied significantly among countries

**Fig. 2:**
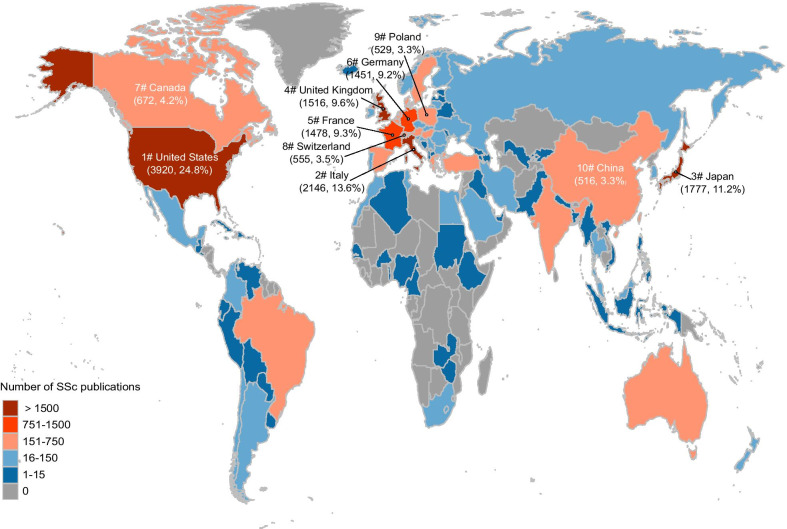
Landscape of SSc publications. Total SSc publication production originating from different countries during 1969–2018 is shown on the world map. Different colors were assigned to countries according to the total number of SSc publications. Warmer colors represent higher SSc publication production and cooler colors represent lower production. Countries without SSc publications were presented in the grey color. The ten countries with the most SSc publications were listed with the rank and number of SSc publications. *SSc* systemic sclerosis

After 2354 publications without country information excluded, the remaining 15 821 journal articles on SSc were attributed to 107 of the 194 WHO member countries (Fig. 2). The remaining 87 WHO member countries producing no SSc publications were mostly located in Africa. Four countries were found with over 1500 SSc publications, while 55 countries produced fewer than 15. The top ten countries produced 77.5% (12 261/15821) of the global SSc publications and were almost all developed countries from North America, Europe, and Asia. Specifically, nearly one fourth (24.8%, 3920/15 821) SSc publications were contributed by the United States. The full list of country production can be found in the Additional file 2: Table S2.

### SSc publication production was associated with country-level factors

Regression analysis on 2000–2017, when the data of all country-level factors were available, was performed to explore the association between these factors and the number of SSc journal publications. Research and development expenditure showed the strongest association with SSc research output especially in MICs (*p* < 0.001; Table 2). Countries with rare disease legislation tended to have more SSc publications (*p* = 0.010), but the effect appeared insignificant in group analysis for countries of high or middle levels, which might be caused by the timing of legislation. Health expenditure was also positively associated with SSc research output (*p* = 0.005) and an even higher association was detected in HICs but not in low to middle-income countries. Population (*p* < 0.001) and GDP (*p* < 0.001) were two structural factors associated with SSc research output (see Additional file 3). However, the effects differed with income groups. The positive association was higher in HICs while a minor negative association without statistical significance was shown in LICs. No association was detected between SSc scientific production and GDP per capita, sex percentage, or governance indicators.

**Table 2:** Associations between country-level factors and SSc scientific output

	All countries	HICs	MICs	LICs
Ln of GDP per capita	0.163 (− 0.012, 0.337)	− 0.028 (− 0.508, 0.451)	0.081 (− 0.104, 0.266)	− 0.043 (− 0.122, 0.035)
Ln of population	0.292*** (0.198, 0.385)	0.534*** (0.379, 0.689)	0.119* (0.016, 0.222)	− 0.017 (− 0.047, 0.013)
Female population percentage	0.004 (− 0.029, 0.037)	0.019 (− 0.041, 0.079)	0.019 (− 0.107, 0.145)	− 0.020 (− 0.057, 0.018)
Voice and accountability	0.152 (− 0.029, 0.334)	− 0.022 (− 0.484, 0.441)	0.179 (− 0.011, 0.370)	0.027 (− 0.026, 0.080)
Government effectiveness	− 0.125 (− 0.346, 0.095)	− 0.329 (− 0.727, 0.068)	0.018 (− 0.234, 0.271)	0.060 (− 0.055, 0.175)
Political stability and absence of violence/terrorism	0.006 (− 0.109, 0.121)	0.125 (− 0.091, 0.342)	− 0.100 (− 0.233, 0.033)	− 0.025 (− 0.075, 0.024)
Research and development expenditure (% of total GDP)	0.526*** (0.292, 0.760)	0.269* (0.046, 0.492)	1.315*** (0.743, 1.887)	− 0.006 (− 0.123, 0.110)
Health expenditure (% of total GDP)	0.073** (0.019, 0.127)	0.142*** (0.059, 0.224)	0.000 (− 0.062, 0.062)	− 0.004 (− 0.014, 0.005)
Rare disease legislation	0.395* (0.094, 0.695)	0.306 (− 0.184, 0.797)	0.061 (− 0.250, 0.373)	NA
Number of countries	132	49	68	15
Number of observations	1442	694	659	89

Panel regression analysis during 2000–2017 assessed association between country level indicators and SSc scientific output measured by ln of SSc publications on all countries with available data and within different income groups. The entries are regression coefficients (95% CI). With the legislation variable, value one was assigned to all countries with rare disease legislation and zero to others. The coefficient of legislation for LICs was omitted for none of the 14 countries had rare disease legislation. Year fixed effects were controlled in all regression analysis

*GDP* gross domestic product, *HICs* high-income countries, *LICs* low-income countries, *MICs* middle-income countries, *SSc* systemic sclerosis

***p < 0.001; **p < 0.01; *p < 0.05

### Rare disease legislation increased SSc scientific productivity

We performed DID analysis over a longer time period of 1969–2018, focusing on the coefficients of the legislation variable $L_{it}$ indicating status of rare disease legislation in a given country $i$ at a given time $t$, to assess the impact of orphan drug legislation more accurately. While only controlling for GDP per capita, population, and female percentage of population, but not fixed effects, Model 1 regression on all 167 countries with available data showed that rare disease legislation increased SSc publication production by 93.7% (95% CI 0.707–1.168; *p* < 0.001; Table 3). The effects remained significant with control for country fixed effects in Model 2 (0.933; 95% CI 0.701–1.165; *p* < 0.001) and additionally year fixed effects in Model 3(0.628; 95% CI 0.390–0.867; *p* < 0.001). The effect can be observed in both HIC (0.443; 95% CI 0.076–0.811; *p* = 0.019) and MIC (0.447; 95% CI 0.051–0.842; *p* = 0.027) groups. Full results with coefficients of covariates (LICs included) were shown in Additional file 4: Table S4. Sensitivity analysis in which missing 2018 data were imputed using 2017 data when available showed similar results (see Additional file 5: Table S5).

**Table 3:** Estimated effects of rare disease legislation on SSc scientific output

	M1	M2	M3
All countries (167 countries, 7649 observations)	0.937*** (0.707, 1.168)	0.933*** (0.701, 1.165)	0.628*** (0.390, 0.867)
HICs (52 countries, 2451 observations)	0.807*** (0.552, 1.062)	0.813*** (0.553, 1.073)	0.443* (0.076, 0.811)
MICs (89 countries, 4026 observations)	0.652*** (0.277, 1.026)	0.640** (0.264, 1.017)	0.447* (0.051, 0.842)
Country fixed effects	Uncontrolled	Controlled	Controlled
Year fixed effects	Uncontrolled	Uncontrolled	Controlled

Panel regression assessed effects of rare disease legislation on SSc scientific output measured by ln of SSc publications. With the legislation dummy variable, value one was assigned to countries from the year after rare disease legislation adoption, and zero to other conditions. Effect heterogeneity among countries of different income levels was evaluated using group analysis. Coefficients of legislation in LICs were not reported, for none of the 26 countries had rare disease legislation. Country covariates available were controlled in all three models (M1–M3). Country fixed effects and year fixed effects were included sequentially in M2 and M3

*HICs* high-income countries, *MICs* middle-income countries, *SSc* systemic sclerosis

***p < 0.001; **p < 0.01; *p < 0.05

**Fig. 3:**
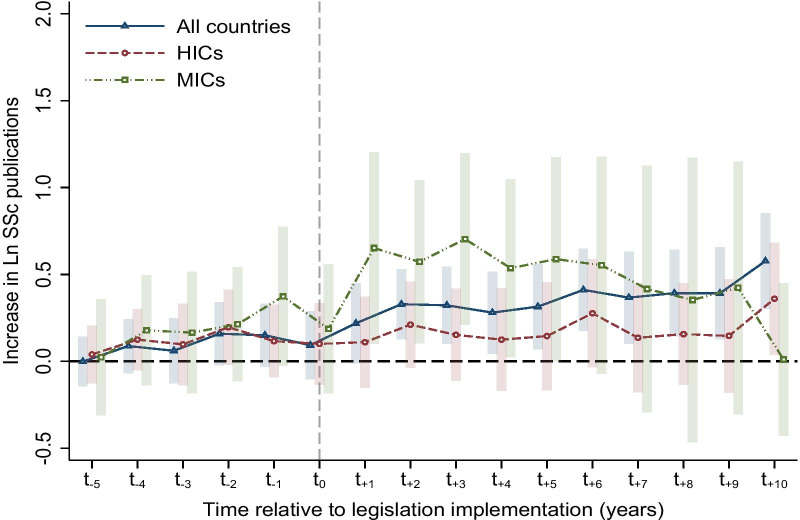
Estimated effects of rare disease legislation on SSc scientific output. Effects of rare disease legislation on ln of SSc publications are presented as regression coefficients (95% CI) separately for all countries (blue), HICs (red) and MICs (green). Legislation dummy variables, t_−5_ to t_+10_ are equal to one in only one year per country with rare disease legislation. t0 refers to the year after legislation implementation. Dummy variables prior t0 (t_−5_ to t_−1_) were used to test for parallel trend, and those after t0 (t_+1_ to t_+10_) showed the dynamics of legislation effect over time. Country and year fixed effects as well as country-level covariates were controlled. *HICs* high-income countries, *MICs* middle-income countries, *SSc* systemic sclerosis

The leads-falsification test confirmed the parallel trend in all included countries as well as in both HIC and MIC groups that countries with or without rare disease legislation shared similar trends of SSc publication output. The effect was shown to be significant and long-lasting with regression on all 167 countries of three income groups, but intriguing differences were shown in the group regression of HICs and MICs (Fig. 3). There was a swift increase of SSc publications the year after implementation of rare disease legislation in MICs, which lasted for at least 5 years but dropped gradually after that. However, no significant effect was observed in the HIC group except on year ten. The full results of the lags and leads analysis are available in the supplementary materials (Additional file 6: Table S6).

## Discussion

This is the first study quantitatively describing the global SSc academic publications and explore the effect of multiple country-level factors. Our study showed that SSc publications increased exponentially with substantial geo-economic inequalities. Rare disease legislation significantly and continually increased SSc publications, especially in MICs. Expenditure on research and development and health was also positively correlated with SSc research output. No positive effects with statistical significance were found with GDP per capita.

SSc publications identified in our study (18,175 during 1969–2018) were substantially fewer than publications identified in studies over diseases with higher prevalence (obesity [7]: 117,340 publications, 1993–2012; Hepatitis B [25]: 49,166 publications, 1971–2011; lung cancer [26]: 32,161 publications, 2004–2013). The low profile of SSc research can be accounted for by the disease rarity but also implied underlying research inefficiency, which was supported by the lagging of increase in comparison to general health and life sciences. Encouragingly, the rate of SSc publication increase in the recent 2 decades exceeded that in general health and life sciences, indicating the state of under-research for SSc is being improved.

On country levels, our results showed SSc publications were mainly from North America, Europe, and Asia, which is consistent with a previous study analyzing the interventional trials on SSc [10]. In contrast, most African countries had no SSc publications. The disadvantageous situation of Africa’s research was also reported on other rare diseases [8, 27]. These results collectively indicated noteworthy between-country inequalities over SSc to be addressed in the future.

The global inequalities might be originated from socio-economic variance. Though economically developed countries played a leading role in SSc research, we found GDP per capita had no significant association with SSc research output. Our results are consistent with most bibliometric studies [13, 28]. These results implied other factors affecting country scientific output. In our study, GDP and population were identified as the two structural factors significantly correlated with country academic output. GDP was also positively related to country total scientific productivity or on other specific topics [13, 28, 29], supporting the logic that larger economies are at a research advantage with more allocable resources, and the speculation that research studies on a rare disease like SSc are challenged with insufficient funding as well as a limited number of patients and researchers [30]. Populous countries are more likely to have more patients, research practitioners, and usually more material resources. These results also indicated that countries with smaller population or economy are at disadvantage in SSc research. More interestingly, the associations were detected to be higher and more significant in HICs than the other two groups, implying that HICs might have more optimal conditions to translate population and economic advantage to research output, which might help to explain the leading role of HICs in SSc research and indicate potential directions for developing countries.

Policy stimulators should be considered as solutions addressing research inefficiency in scientifically disadvantageous countries. We confirmed the significant and long-lasting positive effect of rare disease legislation on SSc publication. The positive effect of rare disease legislation might be attributed to regulatory and economic incentives provided to researchers and pharmaceutical companies [31]. According to our results, rare disease legislation should be adopted by more countries, especially MICs, to promote SSc research. The decreasing of the legislation effect might be associated with the fact that most MICs adopted rare disease legislation only in recent years. Future studies assessing the long-term impact of legislation in MICs may provide additional information.

Expenditure on research and development and health may affect research studies on all biomedical topics through increased investment into science and health. Our regression analysis revealed that expenditure on research and development and health is also associated with increased SSc publications, consistent with studies over other areas [11, 14]. Furthermore, we noticed the association between expenditure and output varied with income groups. MICs might benefit more from research and development expenditure increase rather than health expenditure. More efforts are required to analyze the economic and clinical value of investment into related areas and rare diseases.

In our study, Africa was identified as a key under-researched region. Most African countries were populous but economically disadvantaged LICs, among which we found no significant correlation between country-level factors and SSc research output. Still, our results cannot preclude the potential impact of rare disease legislation, which none LICs have adopted. Technical support and coordinated global efforts are needed to address the research inadequacy of SSc and other rare diseases in Africa, which is also called for by the 17th International Conference on Rare Diseases and Orphan Drugs [32].

There are several limitations in our study warranting notice. Firstly, because our study was carried out on SSc publications in countries with available data, these results may not apply to other rare diseases and countries. However, considering the factors we studied were not specially targeted on SSc research and that at least 2/3 of WHO member countries were included, our results can still provide decision-makers with important information of how country factors affected research output. Secondly, missing data for country-level factors might impair the validity of our results. For example, though revealed to be related to SSc research output, research and development expenditure and health expenditure weren’t included in the DID regression analysis because of imbalanced data missing. Thirdly, there are other factors possibly confounding the results not included due to substantial data gaps, including disease prevalence as well as the proportion of researchers, technicians, doctors, and other relevant practitioners in the population. As an instance, countries more prevailing with SSc might have higher levels of SSc research. However, disease prevalence was not included because of data-scarce and inconsistency resulted from revision of diagnostic criteria in 2013 [33]. Though no validated country patterns of SSc prevalence have been reported, more stringent studies could be carried out when relevant data become available. To minimize possible bias undermining the reliability of our results, we additionally added fixed effects to our DID models (Table 3, Model 2–3) when assessing effect of rare disease legislation, showing that rare disease legislation robustly increased SSc publications. Lastly, it should be noted that having an author contributing to SSc publications does not equal to increasing domestic knowledge on SSc of the particular country. More accurate estimation requires manual filtering and authorship allocating, which is inapplicable in our study for the large number of publications included. Still, the construction of a data panel spanning 50 years using the most recent and reliable data, the inclusion of fixed effects in regression analysis, supplementary tests for parallel trends and sensitivity analysis with imputed data ensured the reliability of the association revealed between country-level factors and SSc research output, especially the causal effect of rare disease legislation.

Overall, our study revealed the increasing pace of SSc publication accumulation in the recent 20 years and points to the substantial imbalance of SSc research distribution among countries. Findings from our study provided evidence concerning national policies for decision-makers to facilitate domestic research and eliminate research inequality.

## Supplementary Information

**Additional file 1**. **Table S1**. Summary of country-level indicators. Definitions, Sources, and year coverage of country indicators included in the study.

**Additional file 2**. **Table S2**. Total number of SSc publications from contributing countries. Lists of contributing countries of SSc publications with numbers of publications and ranks.

**Additional file 3**. **Table S3**. Association between country-level factors and SSc scientific output. Results of additional analyses of panel regression on 2000–2017 including GDP but not GDP per capita and population as the covariate.

**Additional file 4**. **Table S4**. Full results of DID regression models of ln SSc publications. Results of regression analysis on 1969–2018 including coefficients of covariates.

**Additional file 5**. **Table S5**. Sensitivity analysis of DID regression using imputed data. Results of regression analysis on 1969–2018 using imputed 2018 data with 2017 data when available.

**Additional file 6**. **Table S6**. Parallel trends and dynamic effects. Results of regression analysis on 1969–2018 with leading and lagging dummy variables to assess parallel trends and dynamic effects.

## Data Availability

The original list of publications retrieved from Scopus and annual production of publication are available from the corresponding author on reasonable request. Data for country-level factors are available from public sources as cited. Other data are available in the article or supplementary materials.

## Abbreviations

DID: Difference-in-differences
GDP: Gross domestic product
HIC: High-income country
LIC: Low-income country
MIC: Middle-income country
SSc: Systemic sclerosis
