# Supplementary material for: Effects of socio-economic factors on research over systemic sclerosis: an analysis based on long time series of bibliometric data
Source: Orphanet J Rare Dis. 2021 Dec 20;16:517. doi: 10.1186/s13023-021-02149-w (PMC8686627; doi:10.1186/s13023-021-02149-w)
Supplement: Supplementary file 1 — Additional file 1. Table S1. Summary of country-level indicators. Definitions, Sources, and year coverage of country indicators included in the study. [file 13023_2021_2149_MOESM1_ESM.docx]

# Table S1. Summary of country-level indicators

| Indicator | Definition | Source | Year coverage |
| --- | --- | --- | --- |
| GDP | Expenditure-side real GDP at chained PPPs (in mil. 2011US$) | PWT9.1^1^ | 1969–2017 |
| Population | Total population in millions | PWT9.1^1^ | 1969–2017 |
| GDP per capita | GDP divided by population, presented in 2011US$ | PWT9.1^1^ | 1969–2017 |
| Income group (2018–2019) | Country classifications by income level | World Bank Open Data^2^ | NA |
| Female population percentage | The percentage of the population that is female | World Bank Open Data^2^ | 1969–2018 |
| Voice and accountability | Extent of political freedom and participation | WGI3 | 2000–2018 |
| Government effectiveness | Quality of political policies and credibility of government’s commitment | WGI^3^ | 2000–2018 |
| Political stability and absence of violence/terrorism | Degree of political stability and safety | WGI^3^ | 2000–2018 |
| R&D expenditure (% of total GDP) | Gross domestic expenditure on R&D, expressed as a percent of GDP | World Bank Open Data^2^ | 1996–2018 |
| Health expenditure (% of total GDP) | Gross domestic expenditure on health, expressed as a percent of GDP | GHED^4^ | 2000–2017 |
| Rare disease legislation | The status of rare disease legislation | Chan et al.^5^ | 1969–2018 |

The income group indicator was not included as a covariate but used as grouping criteria in regression analysis to show impact heterogeneity in different income groups.

GDP, gross domestic product; GHED, Global Health Expenditure Database; NA, not applicable; PPP, purchasing power parity; PWT, Penn World Table; R&D, research and development; WGI, World Governance Indicators.

1. Feenstra RC, Robert Inklaar and Marcel P. Timmer. The next generation of the Penn world table. Am Econ Rev 2015;105(10):3150–82.
2. Bank W. World Bank. World Bank Open Data. 2019. https://data.worldbank.org/. (accessed 06/26/2020)
3. Kaufmann D, Kraay A. Daniel Kaufmann, Aart Kraays. World Governance Indicators. 2020. http://info.worldbank.org/governance/wgi/. (accessed 07/16/2020)
4. WHO. Global Health Expenditure Database. 2020. https://apps.who.int/nha/database/Home/en. (accessed 08/17/2020)
5. Chan AYL, Chan VKY, Olsson S, et al. Access and unmet needs of orphan drugs in 194 countries and 6 areas: a global policy review with content analysis. Value Health 2020;23(12):1580-91.
