# Supplementary material for: Effects of socio-economic factors on research over systemic sclerosis: an analysis based on long time series of bibliometric data
Source: Orphanet J Rare Dis. 2021 Dec 20;16:517. doi: 10.1186/s13023-021-02149-w (PMC8686627; doi:10.1186/s13023-021-02149-w)
Supplement: Supplementary file 2 — Additional file 2. Table S2. Total number of SSc publications from contributing countries. Lists of contributing countries of SSc publications with numbers of publications and ranks. [file 13023_2021_2149_MOESM2_ESM.docx]

# Table S2. Total number of SSc publications from contributing countries

| Rank | Countries | Number of publications (%) (N =15821) |
| --- | --- | --- |
| 1 | United States | 3920 (24.8%) |
| 2 | Italy | 2146 (13.6%) |
| 3 | Japan | 1777 (11.2%) |
| 4 | United Kingdom | 1515 (9.6%) |
| 5 | France | 1476 (9.3%) |
| 6 | Germany | 1450 (9.2%) |
| 7 | Canada | 672 (4.2%) |
| 8 | Switzerland | 554 (3.5%) |
| 9 | Poland | 529 (3.3%) |
| 10 | China | 516 (3.3%) |
| 11 | Spain | 481 (3%) |
| 12 | Netherlands | 447 (2.8%) |
| 13 | Brazil | 391 (2.5%) |
| 14 | Sweden | 317 (2.0%) |
| 15 | Australia | 295 (1.9%) |
| 16 | Greece and Belgium | 265 (1.7%) |
| 18 | Turkey | 248 (1.6%) |
| 19 | India | 242 (1.5%) |
| 20 | Israel | 241 (1.5%) |
| 21 | Hungary | 233 (1.5%) |
| 22 | Denmark | 191 (1.2%) |
| 23 | Austria | 179 (1.1%) |
| 24 | South Korea | 135 (0.9%) |
| 25 | Mexico | 122 (0.8%) |
| 26 | Czech Republic | 121 (0.8%) |
| 27 | Portugal | 116 (0.7%) |
| 28 | Russian Federation | 113 (0.7%) |
| 29 | Romania | 109 (0.7%) |
| 30 | Norway | 103 (0.7%) |
| 31 | Egypt | 91 (0.6%) |
| 32 | Serbia | 90 (0.6%) |
| 33 | Thailand | 88 (0.6%) |
| 34 | Iran | 77 (0.5%) |
| 35 | Bulgaria | 76 (0.5%) |
| 36 | Argentina | 65 (0.4%) |
| 37 | Ireland | 61 (0.4%) |
| 38 | South Africa | 56 (0.4%) |
| 39 | Croatia | 51 (0.3%) |
| 40 | Colombia and New Zealand | 50 (0.3%) |
| 42 | Finland | 48 (0.3%) |
| 43 | Slovakia | 44 (0.3%) |
| 44 | Singapore | 40 (0.3%) |
| 45 | Tunisia | 35 (0.2%) |
| 46 | Slovenia | 29 (0.2%) |
| 47 | Saudi Arabia | 27 (0.2%) |
| 48 | Malaysia | 19 (0.1%) |
| 49 | Morocco, Chile, Ukraine and Lithuania | 18 (0.1%) |
| 53 | Lebanon | 13 (<0.1%) |
| 54 | Venezuela | 11 (<0.1%) |
| 55 | Dominican Republic, Estonia and Iceland | 9 (<0.1%) |
| 58 | United Arab Emirates, Moldova and Pakistan | 8 (<0.1%) |
| 61 | Bangladesh, Cuba and Malta | 7 (<0.1%) |
| 64 | Nigeria and Iraq | 6 (<0.1%) |
| 66 | Macedonia, Philippines, Senegal and Togo | 5 (<0.1%) |
| 70 | Algeria, Peru, Uruguay and Qatar | 4 (<0.1%) |
| 74 | Cameroon, Guatemala, Kuwait, Nepal and Viet Nam | 3 (<0.1%) |
| 79 | Luxembourg, Albania, Belarus, Cyprus, Indonesia, Jamaica and Zimbabwe | 2 (<0.1%) |
| 86 | Latvia, Armenia, Azerbaijan, Bahrain, Barbados, Bolivia, Bosnia and Herzegovina, Burkina Faso, Ecuador, El Salvador, Ethiopia, Georgia, Grenada, Kiribati, Kyrgyz Republic, Monaco, Myanmar, Paraguay, San Marino, Sudan, Uzbekistan and Zambia | 1 (<0.1%) |

The attribution of publications to countries was based on affiliations and correspondence addresses, with one authorship to each contributing country. The sum of publications from each country exceeded the total number because of co-authorship.
