# Supplementary material for: Effects of socio-economic factors on research over systemic sclerosis: an analysis based on long time series of bibliometric data
Source: Orphanet J Rare Dis. 2021 Dec 20;16:517. doi: 10.1186/s13023-021-02149-w (PMC8686627; doi:10.1186/s13023-021-02149-w)
Supplement: Supplementary file 3 — Additional file 3. Table S3. Association between country-level factors and SSc scientific output. Results of additional analyses of panel regression on 2000–2017 including GDP but not GDP per capita and population as the covariate. [file 13023_2021_2149_MOESM3_ESM.docx]

# Table S3. Association between country-level factors and SSc scientific output (including GDP but not GDP per capita and population)

|  | All countries | HICs | MICs | LICs |
| --- | --- | --- | --- | --- |
| Ln of GDP | 0.266***  (0.175, 0.358) | 0.469***  (0.321, 0.617) | 0.114*  (0.020, 0.207) | −0.017  (−0.046, 0.012) |
| Female population percentage | 0.012  (−0.017, 0.040) | 0.033  (−0.020, 0.087) | 0.015  (−0.111, 0.141) | −0.008  (−0.032, 0.016) |
| Voice and accountability | 0.134  (−0.041, 0.310) | −0.018  (−0.460, 0.424) | 0.179  (−0.011, 0.370) | 0.178  (−0.011, 0.367) |
| Government effectiveness | −0.181  (−0.384, 0.022) | −0.459**  (−0.802, −0.116) | 0.008  (−0.243, 0.260) | 0.046  (−0.047, 0.139) |
| Political stability and absence of violence/terrorism | −0.010  (−0.120, 0.100) | 0.055  (−0.176, 0.286) | −0.103  (−0.230, 0.023) | −0.030  (−0.087, 0.026) |
| R&D expenditure (% of total GDP) | 0.524***  (0.291, 0.757) | 0.252*  (0.037, 0.467) | 1.314***  (0.744, 1.883) | 0.001  (−0.103, 0.106) |
| Health expenditure (% of total GDP) | 0.078**  (0.024, 0.133) | 0.169***  (0.094, 0.244) | 0.002  (−0.061, 0.064) | −0.005  (−0.014, 0.004) |
| Rare disease legislation | 0.343*  (0.053, 0.633) | 0.352  (−0.072, 0.776) | 0.057  (−0.251, 0.365) | NA |
| Number of countries | 132 | 49 | 68 | 15 |
| Number of observations | 1442 | 694 | 659 | 89 |

Regression analysis during 2000–2017 assessed association between country level indicators and SSc scientific output measured on all countries with available data and within different income groups. The entries are regression coefficients (95% CI) based on panel estimation. With the legislation variable, value one was assigned to all countries with rare disease legislation and zero to others. The coefficient of legislation for low income countries was omitted for none of the 14 countries had rare disease legislation. Year fixed effects were controlled in all regression analysis.

GDP, gross domestic product; HICs, high-income countries; MICs, middle-income countries; NA, not applicable; R&D, research and development; SSc, systemic sclerosis

*** p<0.001, ** p<0.01, * p<0.05
