# Supplementary material for: Effects of socio-economic factors on research over systemic sclerosis: an analysis based on long time series of bibliometric data
Source: Orphanet J Rare Dis. 2021 Dec 20;16:517. doi: 10.1186/s13023-021-02149-w (PMC8686627; doi:10.1186/s13023-021-02149-w)
Supplement: Supplementary file 4 — Additional file 4. Table S4. Full results of DID regression models of ln SSc publications. Results of regression analysis on 1969–2018 including coefficients of covariates. [file 13023_2021_2149_MOESM4_ESM.docx]

# Table S4. Full results of DID regression models of ln SSc publications

|  | Rare disease legislation | Ln of population | Ln of GDP per capita | Female population percentage |
| --- | --- | --- | --- | --- |
| All countries (167 countries, 7649 observations): | | | | |
| Model 1 | 0.937***  (0.707, 1.168) | 0.147***  (0.097, 0.197) | 0.281***  (0.186, 0.377) | 0.054***  (0.029, 0.079) |
| Model 2 | 0.933***  (0.701, 1.165) | 0.122**  (0.039, 0.204) | 0.286***  (0.178, 0.395) | 0.055***  (0.024, 0.087) |
| Model 3 | 0.628***  (0.390, 0.867) | −0.701***  (−1.008, −0.395) | 0.054  (−0.036, 0.144) | 0.001  (−0.038, 0.041) |
| HICs (52 countries, 2390 observations): | | | | |
| Model 1 | 0.807***  (0.552, 1.062) | 0.362***  (0.246, 0.478) | 0.528***  (0.311, 0.745) | 0.078***  (0.046, 0.109) |
| Model 2 | 0.813***  (0.553, 1.073) | 0.398*  (0.057, 0.739) | 0.521***  (0.308, 0.734) | 0.095*  (0.022, 0.167) |
| Model 3 | 0.443*  (0.076, 0.811) | −0.697**  (−1.200, −0.195) | −0.130  (−0.412, 0.152) | 0.009  (−0.067, 0.085) |
| MICs (89 countries, 4026 observations): | | | | |
| Model 1 | 0.652***  (0.277, 1.026) | 0.090***  (0.044, 0.135) | 0.246***  (0.123, 0.369) | 0.075***  (0.031, 0.120) |
| Model 2 | 0.640**  (0.264, 1.017) | 0.062  (−0.044, 0.169) | 0.271***  (0.118, 0.425) | 0.098**  (0.039, 0.158) |
| Model 3 | 0.447*  (0.051, 0.842) | −0.411*  (−0.727, −0.095) | 0.135*  (0.016, 0.255) | 0.080**  (0.028, 0.133) |
| LICs (26 countries, 1233 observations): | | | | |
| Model 1 | NA | 0.007  (−0.001, 0.016) | 0.009  (−0.003, 0.022) | 0.019  (−0.012, 0.050) |
| Model 2 | NA | 0.017  (−0.001, 0.034) | 0.006  (−0.003, 0.015) | 0.033  (−0.011, 0.076) |
| Model 3 | NA | 0.006  (−0.058, 0.070) | 0.003  (−0.007, 0.013) | 0.033  (−0.010, 0.076) |

Regression analysis with 7649 observations from 167 countries over the 1969–2018 period assessed effect of rare disease legislation on SSc scientific output measured by ln of SSc publications. The entries are regression coefficients (95% CI) based on panel estimation. With the legislation dummy variable, value one was assigned to countries after one year from rare disease legislation taking effect, and zero to other conditions. All models were adjusted for country level covariates. Missing 2018 demographical and economic data were imputed. Models M2–M3 included country fixed effects and Model 3 included year fixed effects.

NA, not applicable; SSc, systemic sclerosis; GDP, gross domestic production.

*** p<0.001, ** p<0.01, * p<0.05
