# Supplementary material for: Effects of socio-economic factors on research over systemic sclerosis: an analysis based on long time series of bibliometric data
Source: Orphanet J Rare Dis. 2021 Dec 20;16:517. doi: 10.1186/s13023-021-02149-w (PMC8686627; doi:10.1186/s13023-021-02149-w)
Supplement: Supplementary file 5 — Additional file 5. Table S5. Sensitivity analysis of DID regression using imputed data. Results of regression analysis on 1969–2018 using imputed 2018 data with 2017 data when available. [file 13023_2021_2149_MOESM5_ESM.docx]

# Table S5. Sensitivity analysis of DID regression using imputed data

|  | Rare disease legislation | Ln of population | Ln of GDP per capita | Female population percentage |
| --- | --- | --- | --- | --- |
| All countries (167 countries, 7816 observations): | | | | |
| Model 1 | 0.943***  (0.709, 1.178) | 0.153***  (0.101, 0.204) | 0.286***  (0.187, 0.385) | 0.052***  (0.026, 0.078) |
| Model 2 | 0.939***  (0.703, 1.176) | 0.130**  (0.044, 0.215) | 0.291***  (0.178, 0.404) | 0.053**  (0.021, 0.085) |
| Model 3 | 0.633***  (0.389, 0.877) | −0.687***  (−1.006, −0.369) | 0.055  (−0.041, 0.150) | 0.001  (−0.039, 0.041) |
| HICs (52 countries, 2442 observations): | | | | |
| Model 1 | 0.812***  (0.549, 1.075) | 0.379***  (0.261, 0.497) | 0.534***  (0.312, 0.756) | 0.076***  (0.044, 0.108) |
| Model 2 | 0.819***  (0.550, 1.088) | 0.440*  (0.092, 0.788) | 0.523***  (0.306, 0.741) | 0.096*  (0.023, 0.170) |
| Model 3 | 0.442*  (0.063, 0.822) | −0.650*  (−1.167, −0.133) | −0.131  (−0.427, 0.165) | 0.012  (−0.066, 0.091) |
| MICs (89 countries, 4115 observations): | | | | |
| Model 1 | 0.662***  (0.284, 1.041) | 0.092***  (0.046, 0.139) | 0.256***  (0.127, 0.384) | 0.077**  (0.031, 0.123) |
| Model 2 | 0.652**  (0.271, 1.032) | 0.062  (−0.048, 0.173) | 0.281***  (0.121, 0.441) | 0.098**  (0.038, 0.159) |
| Model 3 | 0.460*  (0.060, 0.859) | −0.411*  (−0.736, −0.086) | 0.141*  (0.014, 0.267) | 0.080**  (0.027, 0.134) |
| LICs (26 countries, 1259 observations): | | | | |
| Model 1 | NA | 0.008  (−0.000, 0.017) | 0.009  (−0.002, 0.020) | 0.019  (−0.010, 0.047) |
| Model 2 | NA | 0.018  (−0.001, 0.036) | 0.006  (−0.002, 0.013) | 0.028  (−0.008, 0.065) |
| Model 3 | NA | 0.008  (−0.053, 0.069) | 0.002  (−0.006, 0.011) | 0.029  (−0.006, 0.064) |

Regression analysis with 7816 observations from 167 countries over the 1969–2018 period using imputed 2018 data with 2017 data when available. The entries are regression coefficients (95% CI) based on panel estimation. With the legislation dummy variable, value one was assigned to countries after one year from rare disease legislation taking effect, and zero to other conditions. Models M2–M3 included country fixed effects and Model 3 included year fixed effects.

GDP, gross domestic production; HICs, high-income countries; LICs, low-income countries; MICs, middle-income countries; NA, not applicable; SSc, systemic sclerosis.

*** p<0.001, ** p<0.01, * p<0.05
