# Supplementary material for: Effects of socio-economic factors on research over systemic sclerosis: an analysis based on long time series of bibliometric data
Source: Orphanet J Rare Dis. 2021 Dec 20;16:517. doi: 10.1186/s13023-021-02149-w (PMC8686627; doi:10.1186/s13023-021-02149-w)
Supplement: Supplementary file 6 — Additional file 6. Table S6. Parallel trends and dynamic effects. Results of regression analysis on 1969–2018 with leading and lagging dummy variables to assess parallel trends and dynamic effects. [file 13023_2021_2149_MOESM6_ESM.docx]

# Table S6. Parallel trends and dynamic effects

|  | All countries | HICs | MICs |
| --- | --- | --- | --- |
| Rare disease legislation leads and lags: | | | |
| 5 years prior | −0.011 (−0.056, 0.034) | 0.004 (−0.074, 0.082) | 0.078** (0.026, 0.130) |
| 4 years prior | −0.001 (−0.144, 0.142) | 0.039 (−0.125, 0.203) | 0.024 (−0.310, 0.358) |
| 3 years prior | 0.088 (−0.067, 0.243) | 0.125 (−0.050, 0.300) | 0.178 (−0.139, 0.495) |
| 2 years prior | 0.061 (−0.127, 0.248) | 0.098 (−0.137, 0.332) | 0.165 (−0.184, 0.515) |
| 1 years prior | 0.159 (−0.022, 0.340) | 0.196 (−0.019, 0.411) | 0.213 (−0.114, 0.541) |
| Year on legislation | 0.150 (−0.031, 0.331) | 0.116 (−0.093, 0.325) | 0.374 (−0.026, 0.773) |
| 1 years after | 0.093 (−0.103, 0.289) | 0.101 (−0.134, 0.335) | 0.187 (−0.185, 0.559) |
| 2 years after | 0.219 (−0.011, 0.448) | 0.110 (−0.152, 0.372) | 0.652* (0.101, 1.203) |
| 3 years after | 0.329** (0.128, 0.530) | 0.211 (−0.036, 0.458) | 0.573* (0.104, 1.042) |
| 4 years after | 0.323** (0.100, 0.545) | 0.152 (−0.113, 0.418) | 0.704** (0.211, 1.196) |
| 5 years after | 0.280* (0.044, 0.516) | 0.125 (−0.171, 0.420) | 0.536* (0.024, 1.049) |
| 6 years after | 0.315* (0.071, 0.559) | 0.145 (−0.165, 0.456) | 0.588* (0.002, 1.175) |
| 7 years after | 0.411*** (0.175, 0.647) | 0.277 (−0.033, 0.586) | 0.553 (−0.073, 1.178) |
| 8 years after | 0.367** (0.102, 0.631) | 0.136 (−0.178, 0.449) | 0.416 (−0.292, 1.125) |
| 9 years after | 0.393** (0.145, 0.641) | 0.157 (−0.135, 0.448) | 0.353 (−0.465, 1.172) |
| 10 years after | 0.391** (0.127, 0.656) | 0.146 (−0.180, 0.472) | 0.423 (−0.303, 1.149) |
| Country covariates: | | | |
| Ln of population | −0.922*** (−1.283, −0.561) | −0.867** (−1.382, −0.351) | −0.376* (−0.705, −0.048) |
| Ln of GDP per capita | 0.038 (−0.057, 0.132) | −0.149 (−0.439, 0.140) | 0.133* (0.012, 0.254) |
| Female population percentage | −0.922*** (−1.283, −0.561) | −0.867** (−1.382, −0.351) | −0.376* (−0.705, −0.048) |
| Number of countries | 167 | 52 | 89 |
| Number of observations | 7640 | 2390 | 4026 |

GDP, gross domestic product; HICs, high-income countries; LICs, low-income countries; MICs, middle-income countries; SSc, systemic sclerosis.

*** p<0.001, ** p<0.01, * p<0.05
